# Neuronal migration and its disorders affecting the CA3 region

**DOI:** 10.3389/fncel.2014.00063

**Published:** 2014-03-04

**Authors:** Richard Belvindrah, Marika Nosten-Bertrand, Fiona Francis

**Affiliations:** ^1^INSERM UMR-S 839Paris, France; ^2^Sorbonne Universités, Université Pierre et Marie Curie, Univ Paris 06Paris, France; ^3^Institut du Fer à MoulinParis, France

**Keywords:** neurodevelopment, mouse mutant, epilepsy, lamination, hippocampus

## Abstract

In this review, we focus on CA3 neuronal migration disorders in the rodent. We begin by introducing the main steps of hippocampal development, and we summarize characteristic hippocampal malformations in human. We then describe various mouse mutants showing structural hippocampal defects. Notably, genes identified in human cortical neuronal migration disorders consistently give rise to a CA3 phenotype when mutated in the mouse. We successively describe their molecular, physiological and behavioral phenotypes that together contribute to a better understanding of CA3-dependent functions. We finally discuss potential factors underlying the CA3 vulnerability revealed by these mouse mutants and that may also contribute to other human neurological and psychiatric disorders.

## Basic steps of hippocampal development

The development of the rodent hippocampus in the medial telencephalon starts in mid-embryogenesis, as factors are secreted from the cortical hem to induce, specify and amplify the adjacent neuroepithelium to produce cortical tissue (Hoch et al., 2009; Pierani and Wassef, 2009; Subramanian and Tole, 2009; Subramanian et al., 2009). Hippocampal Cajal Retzius cells are also derived from the cortical hem and required for correct hippocampal organization (Bielle et al., 2005; Chizhikov et al., 2010; reviewed in Khalaf-Nazzal and Francis, 2013). The hippocampus shares a neuroepithelium which extends dorsally to ventrally through neocortical, subicular, hippocampal and septal regions. During hippocampal pyramidal cell neurogenesis, progenitor cells in these neuroepithelial ventricular zones (VZs) divide to produce neurons, which migrate radially toward the pial surface. Specialized progenitors are radial glial cells which have their somata in the VZ, a short apical process descending to the ventricular lining and a long basal process extending up to the pial surface (Nowakowski and Rakic, 1979; Seri et al., 2001). Cajal-Retzius cells in the marginal zone (MZ) play a critical role during development, initially secreting factors, which help maintain radial glial cell morphology and attachment to the pial surface (Zhao et al., 2004).

As shown extensively in the neocortex, radial glial cells can divide either symmetrically or asymmetrically, producing other progenitor cells (basal progenitors) and neurons (reviewed by Götz and Huttner, 2005; Kriegstein et al., 2006). In earlier stages of development, neurons may migrate by somal translocation, moving their nuclei within a long basal process attached to the pial surface (Nadarajah and Parnavelas, 2002). As shown firstly by classical neuroanatomical methods and later by videomicroscopy, radial glial cell processes later serve as guides for migrating neurons (radial glial guided locomotion). A variety of molecules, both intracellular and extracellular, have been shown to influence this mode of migration (see section Mouse Mutants and Molecular Mechanisms Important for Migration and Lamination). Neurons detach from these guides to settle in the cortical plate in a characteristic inside-out lamination pattern (reviewed by Gupta et al., 2002). Somal translocation may also play a role at these end stages of migration. Different waves of migration correspond to neurons born at different timepoints of neurogenesis. Earlier-born neurons settle in the deeper neocortical layers, whilst later born neurons cross through these layers to reach more superficial regions. The exact mechanisms regulating pyramidal neuron detachment from radial glial processes are unclear, although the reelin protein (see section Mouse Mutants and Molecular Mechanisms Important for Migration and Lamination), secreted by Cajal-Retzius cells, may play an instrumental role (Franco et al., 2011). Detached neurons then continue to differentiate, growing their apical and basal dendrites, and long axons.

**Figure 1:**
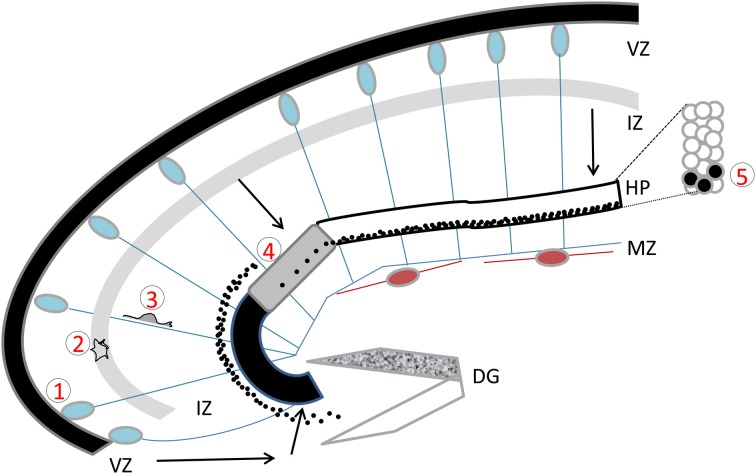
**The developing hippocampus.** Radial glial cells are represented with their somata in the ventricular zone (VZ) and long basal processes extending up to the marginal zone (MZ). These processes serve as guides for migration. Cajal Retzius cells are schematized in brown. Migration pathways across the intermediate zone (IZ) are indicated by arrows. The hippocampal plate (HP) is shown in white, gray, and black to indicate the CA1, CA2, and CA3 fields respectively. The dentate gyrus (DG) is indicated as a V-shaped structure, at this stage the inferior blade (shown in white) is not completely formed, whereas the superior blade (shown in mottled gray) is taking shape. A BrdU injection at E18 in the rat, with sacrifice 4 days later reveals BrdU-labeled cells as schematized by the black dots: cells born at E18 have already reached the CA1 developing pyramidal cell layer, whereas CA3 cells are still found in the IZ, requiring another full day to cross the CA3 pyramidal cell layer. An inset shows the organization of somata in the pyramidal cell layer. Five successive steps of development are indicated, (1) cell proliferation and neurogenesis in the VZ; (2) a multipolar phase above the VZ; (3) a bipolar phase of migration through the IZ; (4) insertion in the hippocampal plate; (5) settling in the appropriate layer. Schema based on data shown in Altman and Bayer (1990b), Figure 3.

Development occurs similarly in the hippocampus. As characterized in detail by Altman and Bayer in the rat dorsal hippocampus (Altman and Bayer, 1990a), using radioactive thymidine to label cells at different ages, radial glial progenitor cells in the convex-shaped ammonic neuroepithelium show a high level of proliferative activity and cycle their nuclei spatially within the VZ in a process termed interkinetic nuclear migration. Waves of migrating cells born at different time-points have also been characterized, however distinct inside-out lamination in the compact hippocampal plate is less well recognized (Altman and Bayer, 1990a; Fleck et al., 2000; Tole and Grove, 2001; Nakahira and Yuasa, 2005). A majority of pyramidal neurons are generated between E16 and E20 in the rat hippocampus. By E20/E21 there are many fewer cells proliferating in the ammonic neuroepithelium. Migrating neurons in the hippocampal intermediate zone (IZ) were recognized as scattered spindle-shaped cells distinguishable from periventricular mitotic cells. In a second paper by the same authors (Altman and Bayer, 1990b), migrating cells were found to organize in bands, one more compact closer to the neuroepithelium and one more diffuse closer to the forming pyramidal cell layer. Cells were found to settle in the pyramidal cell layer (or hippocampal plate) 3–5 days after leaving the neuroepithelium (Figure 1), after a pause period, which the authors termed “sojourning.” Sojourning has been proposed to allow pyramidal neurons to begin to polarize and to initiate axon growth which may occur far from the hippocampal plate (Altman and Bayer, 1990b). As post-mitotic neurons leave the VZs, they first take on a transient “multipolar” morphology, moving small distances laterally in the sub-VZ, whilst potentially waiting for appropriate signals which will induce them to acquire a bi-polar morphology with a leading and trailing process, perpendicularly oriented with respect to the ventricular lining. These morphological changes have been nicely characterized using the technique of *in utero* electroporation to label isolated cells focally with a fluorescent marker (Nakahira and Yuasa, 2005; Navarro-Quiroga et al., 2007).

Nowakowski and Rakic (1979), studying migration in the fetal monkey hippocampus, also extensively characterized this radial-glial guided migration, but no mention was made of other migration modes. Even concerning the end stages of migration, as cells reached the hippocampal plate, although these authors recognized less dependence on radial glial cells and more complex multipolar morphologies, no mention was made of somal translocation. This is also true for the more recent studies in the rodent using immunofluorescence to reveal cell morphologies (Manent et al., 2005; Nakahira and Yuasa, 2005; Navarro-Quiroga et al., 2007). Nowakowski and Rakic do cite Morest (1970), studying the opossum brain, who identified an outer process extending through the hippocampal plate from the time the cell somata leave the VZ until they attain their final position. It is unclear if this data refers to somally translocating cells, or radial glial cell guided migration. From these studies, it is hence not yet possible to conclude if somal translocation is used in the hippocampus. Instead, another mode of migration, the “climbing mode” has recently been characterized. This occurs at late stages of cellular integration in the pyramidal cell layer, when migrating neurons are observed to exhibit multiple highly branched neuronal processes which interact with a number of radial fibers. Neurons are seen to switch radial fibers regularly, advancing in a zigzag manner through the hippocampal plate (Kitazawa et al., 2014). This mode of migration has never been characterized in the neocortex, may be specific to the hippocampus and may contribute to its less distinct lamination.

The pyramidal cell layer grows progressively from the subiculum to the dentate gyrus (DG), with the CA1 formed before the CA3 region. Studies in the rodent show that the migration time was shorter for CA1 pyramidal cells (4 days) than CA3 (5 days), although migration to both these regions is slower than cortical neurons migrating in the neocortex (Altman and Bayer, 1990b; Nakahira and Yuasa, 2005). Thus the CA1 layer becomes visible before the CA3, although its peak of neurogenesis (E18 in the rat) is a day later than the CA3 region (E17 in the rat). In the gradient of neurogenesis from the subiculum to the DG, CA1 neurogenesis is noticeably retarded and this has been associated with the requirement for thalamic inputs (Bayer, 1980). Alternatively, there is cumulative evidence for coordinated development and connectivity of different neuronal types within and outside the hippocampus (Bayer, 1980; Altman and Bayer, 1990b; Manent et al., 2005, 2006; Deguchi et al., 2011) which might help explain the comparatively longer “sojourning” time for CA3 pyramidal cells, which need to coordinate with DG granule cells. The curvature of the CA3 region and complexity of the migration path are also likely to contribute to this phenomenon. Indeed, unlike the CA1 region, CA3 cells not only have to migrate further, but they form a pyramidal cell layer which curves away from the neuroepithelium toward the DG. There is also a gradient of production of cells within the CA3 region itself, the regions closer to the CA1 region (CA3a and b subdivisions) forming before the region closest to the DG, and dorsal CA3 forming before ventral (Bayer, 1980). Indeed, CA3 cells destined for the hilar region (CA3c subdivision) have the furthest to travel, and they are eventually framed by the DG blades. Particular characteristics of radial glial cells at this stage of development are likely to be instrumental, playing a role as a substrate to guide CA3 cells to this destination. DG cells may use the same migratory path as they migrate tangentially from the dentate neuroepithelium to the DG region (Altman and Bayer, 1990a,b; Nakahira and Yuasa, 2005; Danglot et al., 2006; Barry et al., 2008; Khalaf-Nazzal and Francis, 2013). Barry et al. (2008) have shown that RG cells progressively express different markers, and the formation of the DG (and most probably the hilar CA3) region is particularly dependent on a radial glial subtype present at later stages of development, extending from the neuroepithelium to the DG region. This represents a difference compared to the CA1 region, which can still form in the presence of “earlier” RG cells. There is also a less distinct deep to superficial lamination in regions of the CA3c layer, with these cells, although grouped together, showing a certain amount of spreading compared to other CA regions (Bayer, 1980). All in all, and despite the shorter migration distances, the pattern of development and morphogenesis of the hippocampus seems relatively more complicated than the formation of the neocortex, related to its fields of different shapes and compositions, with the CA3 pyramidal cell layer being more complex than the CA1 in this respect. Cell heterogeneity within each of the CA1 and CA3 regions is also now a well-recognized phenomenon (Thompson et al., 2008; Dong et al., 2009; Christian et al., 2011; Slomianka et al., 2011; Graves et al., 2012; Nielsen et al., 2013). Time of birth, migration pathways, as well as extracellular factors all contribute to different cell identities in the adult.

Elizabeth Grove and colleagues characterized genetic markers distinguishing CA1 and CA3 fields, appearing during development (Tole et al., 1997). Many markers are now known in the adult, related to large-scale *in situ* hybridization data in the mouse (Thompson et al., 2008) which have revealed nine different subfields for the CA3 region, showing a remarkable relationship with different functional connectivity boundaries. Laser microdissection transcriptome studies are also adding to the identification of CA3-specific markers (Datson et al., 2004, 2009; Greene et al., 2009; Deguchi et al., 2011), which may in the future reveal novel developmental gene networks playing key roles specifically in CA3 migration and differentiation.

While the settling of pyramidal neurons in the CA regions occurs through local migration from the ammonic neuroepithelium, hippocampal interneurons have to migrate very long distances from their places of origin to their final destination in the hippocampus (reviewed by Danglot et al., 2006). Hippocampal interneurons destined for the CA region are produced in the ventral telencephalon of the mouse from E12 onwards (Soriano et al., 1986, 1989; Pleasure et al., 2000; Manent et al., 2006). When the transcription factor genes *Dlx*1 and 2 are inactivated, the migration of interneuron precursors is completely arrested in the subpallium, related to an upregulation of a cytoskeleton regulator, p21-activated serine/threonine kinase, PAK3 (Anderson et al., 1997; Cobos et al., 2007). Interneurons are indeed derived from the medial (MGE) and the caudal ganglionic eminences (CGE), which generate interneurons sequentially in two waves at E9–E12 and E12–E16, respectively (Anderson et al., 1997; Marin et al., 2001; Danglot et al., 2006). The first interneurons arrive in the hippocampus from E14 onwards. Interneurons use a tangential mode of migration to reach the pallium, migrating in streams, and progressively invading the hippocampus through the MZ and the IZ/subventricular zone (SVZ), with the MZ contributing the vast majority of interneurons (Manent et al., 2006). They terminate migration locally, with MGE interneurons reaching the *strata pyramidale* and *oriens*, and CGE interneurons arriving in the superficial *strata lacunosum moleculare* and *radiatum* (Tricoire et al., 2011). Although the routes of migration are clearly defined, the substrate of migration of interneuron precursors is not. It has been suggested that corticofugal axons might serve as a scaffold for interneuron migration by recruiting the adhesion molecule TAG-1 (Denaxa et al., 2001), but this remains controversial and needs to be further clarified (Denaxa et al., 2005). Similarly, while it has been proposed that radial glia could also constitute guides for interneuron migration final positioning (Polleux et al., 2002; Poluch and Juliano, 2007), these observations lack molecular evidence and still need to be explored. Because of the important diversity of interneurons in the CA fields (Fishell and Rudy, 2011), different approaches of genetic fate mapping have successfully correlated the precise location of neurogenesis in the subpallium with the generated subtype. Mice carrying a GFP transgene either under the control of the *Gad*65 or *Nkx*2-1 promoters, allowed a specific tracking of the fate of interneuron precursors from the CGE or MGE respectively (Tricoire et al., 2011). There exists a striking association between the origin of interneurons, their respective markers and their electrophysiological properties. For instance, precursors originating from the MGE generate parvalbumin^+^, somatostatin^+^, and nNOS^+^ interneurons with specific individual electrophysiological signatures, while the CGE-generated interneurons are cholecystokinin^+^, calretinin^+^, vasoactive intestinal peptide (VIP)^+^ and reelin^+^, each with distinct properties (Tricoire et al., 2011). These markers appear relatively homogenously distributed in the different strata constituting each of the CA fields, although subtle differences in number, morphology and layer distribution are recognized (Freund and Buzsaki, 1996; Matyas et al., 2004). For instance, while parvalbumin^+^, calretinin^+^, and neuropeptide Y^+^ interneurons are mainly located in the *stratum pyramidale* in the CA1 region, they are distributed throughout all strata of the CA3 region. Also, VIP^+^ interneurons seem to be more abundant in CA1 than in CA3 (Freund and Buzsaki, 1996; Matyas et al., 2004). The impact of these and other differences on CA1 vs. CA3 pyramidal cell functions remains to be completely explored.

All in all each of these steps of hippocampal development are instrumental to create the specific identities of the fields. Fundamental molecular, anatomical, and structural network differences between CA1 and CA3 regions suggest that these pyramidal cells have distinct functions. For instance, the recurrent network related to CA3 pyramidal cells differs greatly from the feed-forward network of the CA1 region. Functional intrinsic properties however, that might distinguish these pyramidal cell populations have not however been easily identified between CA1 and CA3 pyramidal cells (for example, Paz-Villagrán et al., 2004). It is possible that such differences at the single cell level could be very small. It has hence not been possible to ascertain the impact of potentially specific intrinsic properties compared to network effects on their functions. However, recently *in vivo* recordings from large sets of CA1 and CA3 pyramidal cells under various brain states and in different environments, clearly demonstrated significant differences in firing rates, spike burst propensity, spike entrainment by theta rhythm, and other spiking dynamics in a brain state-dependent manner (Mizuseki et al., 2012). Studies such as this are hence revealing functional measures to distinguish the two cell populations. Thus, CA1 and CA3 pyramidal cells seem to exhibit specific activity dynamics that may support their distinct computational roles.

## Human hippocampal development and disorders

As mentioned above, radial glial guided migration has been characterized in the fetal monkey hippocampus, suggesting that in primate, hippocampal development occurs in a similar fashion to rodent (Nowakowski and Rakic, 1979). The different steps of human hippocampal development have been characterized (Arnold and Trojanowski, 1996; Abraham et al., 2004) by immunohistochemistry experiments using fetal tissue. At 10 gestational weeks (GW), Abraham et al. described that the compact ammonic plate was not yet visible, but many proliferating cells were observed in the ammonic VZ. Cajal-Retzius cells were also observed in the MZ at this stage. Between 11 and 16 GW, the CA and DG fields became obvious and vimentin staining revealed radial glial like cells in the neuroepithelia, extending from the VZ to the pial surface (Abraham et al., 2004). In this same study, reelin positive interneurons were also identified in the MZ above the hippocampal plate from 14 GW onwards. Proliferating cells become obvious in the dentate matrix from 14 GW onwards and had receded by 21 GW, when a tertiary matrix had formed in the hilar region. These cells had distributed to the DG region by 16 GW. Quantitative Golgi studies have also revealed that dendritic arborization, spine development and synaptogenesis of pyramidal neurons (CA3) occur during the second and third trimesters, and may continue throughout childhood (Lu et al., 2013). By 32–34 GW, it was described that neurons in CA2 and CA3 had undergone rapid enlargement and morphologic maturation, surpassing CA1, which still contained some immature neurons (Arnold and Trojanowski, 1996). Histological data has also been compared to 7-Tesla magnetic resonance imaging (MRI) data, which allowed a high resolution visualization of the different hippocampal fields in post-mortem fetal tissue (Milesi et al., 2014). These results are useful as a reference for the identification of human hippocampal abnormalities.

Hippocampal abnormalities have been found in association with a number of brain malformations, including agenesis of the corpus callosum, lissencephaly, holoprosencephaly, Fukuyama muscular dystrophy, polymicrogyria, heterotopia, focal cortical dysplasia, tuberous sclerosis, and schizencephaly (Sato et al., 2001; Porter et al., 2003; Montenegro et al., 2006; Kappeler et al., 2007; Fallet-Bianco et al., 2008; Donmez et al., 2009; Kuchukhidze et al., 2010). Such hippocampal defects in the past may have often been overshadowed by severe neocortical defects and not well-detailed due to the resolution of the MRI. It has more recently been shown in large cohorts that 30–55% of patients with cortical defects show hippocampal abnormalities (Donmez et al., 2009; Kuchukhidze et al., 2010). In these studies, bilateral and diffuse malformations were most often associated with such hippocampal abnormalities. Defects vary from small hippocampi and hypoplasia, enlarged hippocampi, to abnormally oriented hippocampi, related to abnormal gyration or infolding (Sato et al., 2001). Abnormal atrophy and severe neuronal loss can also be observed. Similar abnormalities are sometimes observed in febrile seizure, partial and refractory epilepsy patients, with no other obvious cortical abnormalities (Porter et al., 2003). Among cortical development disorders, those related to neuronal migration are the most highly associated with hippocampal abnormalities, as reported for lissencephaly (78%) (Donmez et al., 2009). In two neuropathological studies of type 1 lissencephaly cases, the pyramidal cell layers were found to be diffuse, in some cases the CA and DG fields were difficult to distinguish, and a general hypoplasia was observed (Kappeler et al., 2007; Fallet-Bianco et al., 2008). As mentioned below, hippocampal defects are a signature of mice which are mutant for these type I lissencephaly genes (Khalaf-Nazzal and Francis, 2013). This suggests that some genes play conserved functions during hippocampal neuron migration in rodent and primate.

## Mouse mutants and molecular mechanisms important for migration and lamination

The pyramidal cell layer is crudely laminated along the radial axis, with deep to superficial, molecular, morphological and connectivity specificities (Altman and Bayer, 1990a,b,c; Thompson et al., 2008; Deguchi et al., 2011; Slomianka et al., 2011). Thus pyramidal cells are heterogeneous, their birth-date determines their final position, and both the timing of neurogenesis and external factors contribute to their identity. Concerning cell positioning, migratory mechanisms are finely tuned in different cell autonomous and non-cell-autonomous ways and play a key role for the appropriate development and lamination of the CA3 region. At the level of the individual cell, settled in the pyramidal cell layer, it is also sequentially connected by afferent fibers, which also follow a strict temporal and spatial order (Bayer, 1980; reviewed by Förster et al., 2006). Lamination, at both the somal and connectivity levels, may hence be critical for function.

### Cell autonomous mechanisms

The main cell intrinsic actors known to regulate the migratory activity of pyramidal cell precursors are major components of microtubule (MT) structures, or proteins linked to MTs, such as Microtubule Associated Proteins (MAPs) or MT motors. They regulate different aspects of saltatory cellular migration movements such as the elongation of the leading process or nuclear translocation. Some of these regulators, which are part of a generic program that commonly regulates neuronal migration in different cerebral regions such as the cortex, the cerebellum or the hippocampus, are also tightly regulated by region-specific cell to cell signaling.

**Figure 2:**
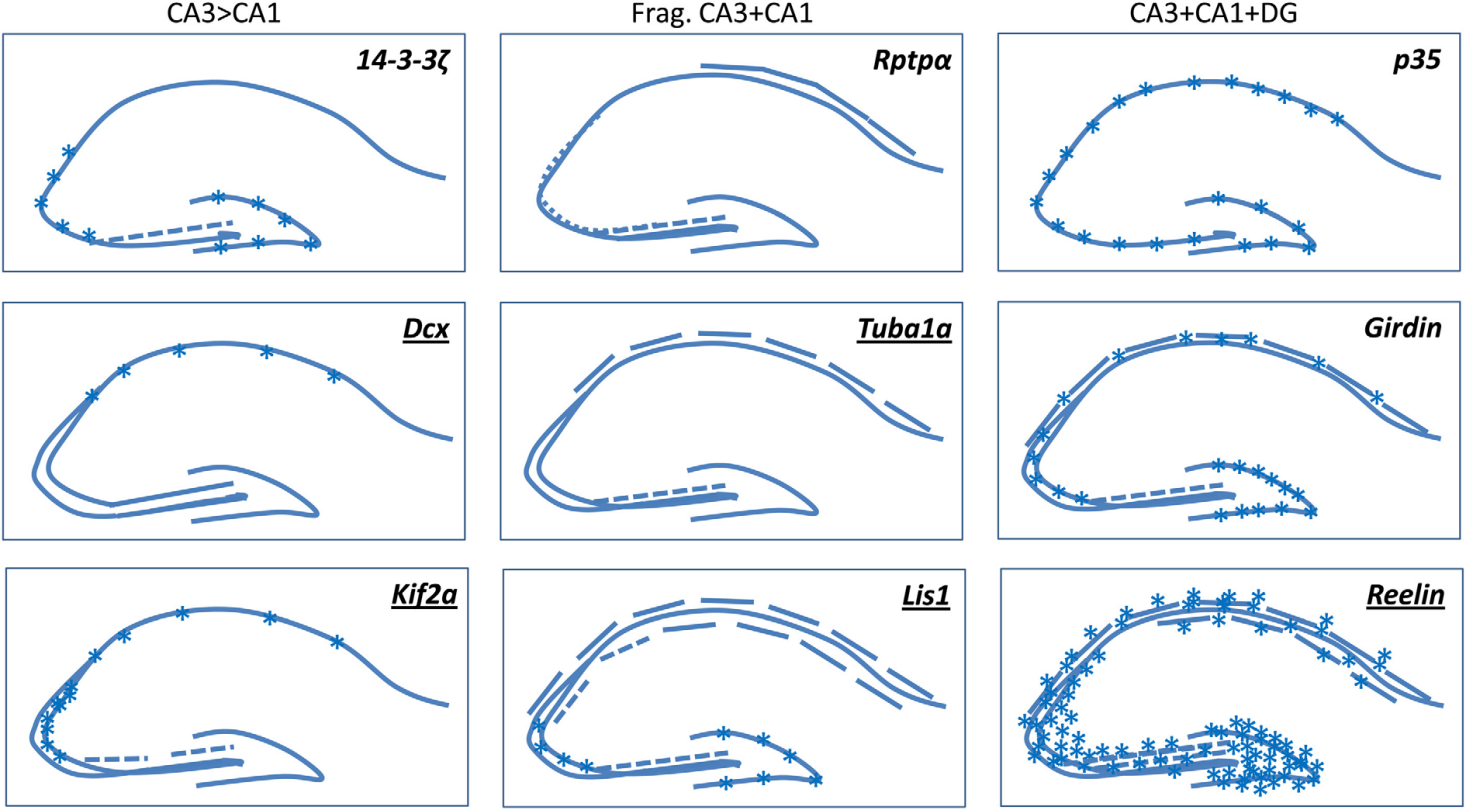
**A selection of mouse mutants showing somal lamination defects in the hippocampus.** Underlined genes are known to be involved in cortical malformations in human. Frag, Fragmented. Broken lines indicate degrees of fragmentation. Asterisks indicate either heterotopic cells or diffusely packed cells. Schemas based on images presented in the following papers: (Del Río et al., 1997; Fleck et al., 2000; Wenzel et al., 2001; Corbo et al., 2002; Homma et al., 2003; Petrone et al., 2003; Kappeler et al., 2007; Keays et al., 2007; Enomoto et al., 2009; Cheah et al., 2012). *Kif*2a mutants die at birth, the P0 images shown in Homma et al. (2003) are similar to *Dcx*-KO brains, we have hence extrapolated these data in our schema depicting adult brains.

Several mouse mutant lines for either tubulin or MT associated components present severe defects in the CA3 region. The ENU-induced mouse mutant showing a mutation in *tubulin*-*alpha1a* (*Tuba1a*) presents a severe fragmentation of the CA layers, including the CA3 region (Keays et al., 2007, see Figure 2 for schemas). The S140G mutation in Tuba1a might generate a haploinsufficiency phenotype with a defect in the incorporation of this mutant tubulin in MTs, which in turn is likely to perturb MT growth rate and consequently migration. Following the identification of this mutant gene in the mouse, mutations were identified in *TUBA1A* in type 1 lissencephaly in human (Keays et al., 2007). Despite the importance of structural homology with several other existing tubulin isotypes in migrating neurons, these results reveal the unique properties of some individual tubulins.

When the expression of certain MAPs is perturbed in mice, similar phenotypes have been observed. Among them, the Doublecortin (Dcx) MAP (Francis et al., 1999; Gleeson et al., 1999), also involved in type 1 lissencephaly, when inactivated in mouse generates a striking anatomical defect, mainly restricted in the CA3 region, with an abnormal double layer of pyramidal neurons. Interestingly, partners of Dcx, spinophilin and Usp9x, when inactivated in the mouse give a similar phenotype (Friocourt et al., 2005; Bielas et al., 2007; Stegeman et al., 2013). The origin of the defects is likely an altered migration although the exact perturbed mechanisms have yet to be fully characterized (Corbo et al., 2002; Kappeler et al., 2007; Nosten-Bertrand et al., 2008; Bazelot et al., 2012). Comparing with what is known for cortical pyramidal neurons and during interneuron migration, Dcx is likely to control neuronal migration by regulating branching and elongation of the leading process, and nuclear translocation, by stabilizing MT structures (Bai et al., 2003; Kappeler et al., 2006; Koizumi et al., 2006a; Belvindrah et al., 2011). The mouse model in particular has been extensively queried for CA3 functions (see “Electrophysiological, Behavioral, and Cognitive Phenotypes” section). Other members of the Dcx family also contribute to radial neuronal migration. *Doublecortin-like kinase* 1 (*Dclk*1) mutants show no obvious hippocampal abnormalities (Deuel et al., 2006; Koizumi et al., 2006b), however, double knockouts for Dcx and Dclk1, show both neocortical and hippocampal defects, conserving a severe fragmentation of the CA3 layer (Deuel et al., 2006). Mice deficient for both *Dcx* and *Dclk*2 show CA1 and CA3 lamination defects, without neocortical defects, and a reduced packing density of cells in the DG, as well as spontaneous seizures (Kerjan et al., 2009). Thus Dclk1 and 2 contribute to neuronal migration in other regions, although Dcx itself seems to have a primary importance in the CA3 region.

Another important MT regulator is the lissencephaly protein Lis1 that interacts within a multiple protein complex formed by dynactin/dynein and Ndel1. *Lis*1 mouse mutants (Hirotsune et al., 1998) present severe defects in hippocampal development such as the presence of heterotopic pyramidal cells in both CA1 and CA3 regions, forming multiple distinct fragmented cell layers (Fleck et al., 2000), as well as granule cell dispersion and aberrant neurogenesis in the DG (Wang and Baraban, 2007, 2008; Hunt et al., 2012). Lis1 by interacting with Ndel1 and dynein participates in a dose-dependent manner to the coupling of the centrosome with the nucleus, an essential step that synchronizes the nuclear movement with the elongation of the leading process (Hirotsune et al., 1998; Shu et al., 2004). Lis1 also contributes to neuronal process stabilization *per se* during migration by interacting with MT motors (Sasaki et al., 2000; Smith et al., 2000). Lis1 and Dcx may act synergistically by regulating dynein function to mediate the coupling of the centrosome to the nucleus (Tanaka et al., 2004a) or by influencing MT stabilization and bundling (Sapir et al., 1997; Francis et al., 1999; Gleeson et al., 1999). Perhaps related to this partial functional overlap, *Lis*1 and *Dcx* mutants present similarities in their respective pyramidal cell phenotypes but, interestingly, also noteworthy differences. While pyramidal cell somata are heterotopic and are less densely packed in both CA1 and CA3 in *Lis*1 mutant mice (Fleck et al., 2000), *Dcx* mutants present mainly defects in the CA3 region, evoking once again possible divergent redundancies at least for the CA3 region, between these molecules.

The MT motor Kif2a is a further example of a protein with a MT function, which is required for hippocampal pyramidal cell organization in the mouse (Homma et al., 2003) and neocortical development in human (Poirier et al., 2013). This protein regulates MT dynamics by depolymerizing MTs. Mutant mice show a hippocampal phenotype, which is similar to *Dcx* mutants, the CA3 region is divided into two layers, and a thin heterotopic band is also seen during development in the CA1 region (Homma et al., 2003). The morphology of cells after settling in the CA1 pyramidal layer was analyzed in these mutants revealing an excess of axon collateral branching. This protein is likely to have pleiotropic functions, and an excess of branching may similarly perturb neuronal migration, as has been observed in *Dcx* mutant cells (Kappeler et al., 2006; Koizumi et al., 2006a; Belvindrah et al., 2011). A fragmented CA3 region is also seen in *14-3-3*ζ mutant mice, once again resembling *Dcx* and *Kif2a* mutants (Cheah et al., 2012). It will be interesting to search for physical interactions between these intracellular proteins, likely to act in the same pathways in CA3 cells.

An important regulator of cytoskeletal proteins is Cyclin Dependent Kinase-5 (Cdk5). Cdk5 mediates phosphorylation of Neurofilament proteins, Map1b, Map2 as well as the MAP Tau. Genetic alteration of its expression (or its activator p35) revealed its important function in neuronal migration since the mutation generates a lack of a confined layer of pyramidal neurons in CA regions (Ohshima et al., 1996; Wenzel et al., 2001; Ohshima et al., 2007). Pyramidal neurons in both CA1 and CA3 are less densely packed and interrupted in places (Chae et al., 1997). Among other Cdk5 substrates are Nudel, a Lis1 interacting protein (Niethammer et al., 2000), Dcx (Tanaka et al., 2004b), Fak (Xie et al., 2003) and Disc-1, a protein associated with the centrosome and MTs (Ishizuka et al., 2011). Thus, Cdk5 fulfills a range of functions extending from nuclear translocation to remodeling of the cytoskeleton by regulating MT dynamics, and its function is critical for neocortical and hippocampal development.

Most of the cytoskeletal components shown to be important for neuronal migration are either intrinsic to MTs or are related to proteins associated with MTs. MT structures are thus required for the dynamics of the anterior part of the leading process, the elongation of the leading process *per se* and the remodeling of the perinuclear cage structure. How the different actin regulators also participate during neuronal migration, in synergy with the MT components, is not completely understood, even for the well studied migration of excitatory neurons in the neocortex. However, the function of the actin cytoskeleton has been revealed at the leading edge of the growth cone, where actin polymerization is finely tuned by Rho-GTPases, such as RhoA (likely regulated by p27Kip1, Kawauchi et al., 2006; Nguyen et al., 2006) and Rac1 (Kholmanskikh et al., 2003). The actin cytoskeleton is also important at different focal contacts initiated in part by N-cadherins (Franco et al., 2011), where adhesions are formed, stabilized or removed. Moreover, the Rho effectors such as mDia and ROCK, interacting with the actin filaments located at the rear of the nucleus promote the actomyosin bundles required for nuclear translocation (Shinohara et al., 2012). There is also molecular evidence of cross interactions between the MT and actin cytoskeletons, and of interest here, certain mouse mutants show hippocampal malformations. Among them, we can identify the Lis1/Cdc42/Iqgap1 complex where Lis1 could increase active Cdc42 and connect the plus-end MT to cortical actin required for neuronal motility (Kholmanskikh et al., 2006). Girdin is also an actin-binding protein which interacts with Disc-1, itself interacting with the MT cytoskeleton (Enomoto et al., 2009). Girdin mouse mutants show hippocampal defects (see below). More recently, a conditional mouse mutant for the MT-actin crosslinking factor 1a (Macf1), that is part of the plakin family and is supposed to bridge MTs with the actin meshwork, also revealed the importance of these regulatory cross-talks, since the mutants present single-band heterotopias in the CA1-CA3 regions (Goryunov et al., 2010). Thus correct functioning of the actin cytoskeleton seems clearly also critical for hippocampal cell migration.

One further similar mouse mutant, *hippocampal lamination defect* (*Hld*, Nowakowski and Davis, 1985), which arose spontaneously on the BALBc background, has also been described although to our knowledge the mutant gene (autosomal) is still unknown. In this mutant, the CA3c region was shown to be inverted, with later-generated neurons found in the deeper instead of superficial layers. This situation could also be true for certain of the above-mentioned mutants and the consequences of such an inversion on function are still unknown.

### Non-cell-autonomous mechanisms

Cell autonomous processes are also entry points for specific modulation of neuronal migration by environmental cues provided by neighboring cells that could be Cajal-Retzius cells, radial glial cells, migrating interneurons or the migrating pyramidal cells themselves, as well as incoming mossy fibers for CA3 cells. Thus, despite the existence of an intrinsic program that defines subtype specificity according to the timing of neurogenesis (Deguchi et al., 2011), the overall architecture of a layered CA3 region is also determined by the cellular integration of complex environmental cues.

As mentioned previously, maintenance of radial glial scaffold integrity is crucial for appropriate glial-guided locomotion of pyramidal neurons. Different mouse mutant models present severe defects in neuronal migration in the CA regions when these glial fibers are disorganized. As in the neocortex, when these fibers lose their apical attachment or their parallel orientation, neuronal migration is strongly compromised. Components of the extracellular matrix are known to regulate neuronal migration during CA3 development. The 385 kDa protein reelin, a major protein secreted by Cajal Retzius cells and shown to control cell positioning in the neocortex, is also implicated in the migratory mechanism of CA1 and CA3 pyramidal neurons. Reelin interacts with its receptors ApoER2/VLDLR to regulate intracellular effectors such as Dab1 (Hiesberger et al., 1999). Mutants for *reelin* (Stanfield and Cowan, 1979b; Niu et al., 2004) and *ApoEr2*/*Vldlr* (Trommsdorff et al., 1999) or their cytoplasmic effector *Dab*1 (Rice et al., 1998), present severe hippocampal development defects, among them, an absence of formation of a tight layer of pyramidal neurons, with cells only loosely organized.

Since the radial glial scaffold is also severely perturbed in these *reelin* signaling hippocampal mutants (Forster et al., 2002; Weiss et al., 2003), the contribution of this signaling pathway to different aspects of neuronal migration is certainly multiple, and this is very likely to be a convergence point between non-cell-autonomous and cell-autonomous regulators. For example, secreted reelin can control radial glial scaffold development, however it can also influence interaction of phosphorylated Dab1 with Lis1 within migrating neurons (Assadi et al., 2003; Zhang et al., 2007). The severe phenotype observed in Lis1 mutants could also be the consequence of a deregulation of the reelin signaling pathway within these neurons themselves. The other cell intrinsic regulator of neuronal migration, Cdk5, also converges with Reelin signaling (Ohshima et al., 2001; Beffert et al., 2004).

Another important regulator of cell migration in the CA3 region is the Receptor Protein Tyrosine Phosphatase, Rptp-α (gene name *Ptpra*). While *Rptp*-α mouse mutants present neuronal migration defects in CA regions, the primary cause of these defects is still unclear. Rptp-α has been shown to be important in the formation of apical dendrites (Ye et al., 2011). The Rptp-α signaling pathway that activates Fyn and Src kinases might also contribute to the proper development of the radial glial scaffold for promoting appropriate neuronal migration, but in this case, this would be through a mechanism independent of reelin signaling (Petrone et al., 2003). More recently, mutants for *Nuclear Factor Ib* (*Nfib*) have also confirmed the importance of the integrity of the glial scaffold to provide the appropriate substrate for neuronal migration. Indeed, in these mutants the glial fibers originating in the ammonic neuroepithelium may fail to mature, compromising severely the development of the hippocampal structure, especially in the DG, and potentially also CA3 subregions (Barry et al., 2008).

The proper migration stimulatory cues for pyramidal neurons in the CA3 region are also provided by migratory interneurons through non-vesicular release of GABA and likely by the migratory pyramidal cells themselves, with glutamate release stimulating the migration through the activation of NMDA receptors (Manent et al., 2005). Thus multiple factors and multiple membrane proteins are likely to influence migration. The sensitivity of migrating neurons to extracellular cues can also be modulated by the turn-over of membrane receptors at the level of the neuronal growth cone. Intracellular factors such as MTs and MAPs are also likely to play a role in this process, adjusting the sensitivity to such extrinsic parameters by modulating the levels of proteins on the surface of the cell by endocytosis and recycling, as has been suggested for Dcx (Friocourt et al., 2001; Kizhatil et al., 2002; Yap et al., 2012).

### Non-genetic mechanisms perturbing CA migration

Up till now we have mentioned genetic factors leading to perturbed CA cell migration. Other factors can lead to this phenotype including injection of methylazoxymethanol (MAM) a DNA alkylating agent (Cattabeni and Di Luca, 1997; Colacitti et al., 1999; Battaglia et al., 2003). This agent when injected in pregnant female rats or mice leads to brain malformations in the embryos, associated with perturbed hippocampal development and susceptibility to seizures (Chevassus-Au-Louis et al., 1998a,b; Colacitti et al., 1999; Baraban et al., 2000). Focal heterotopias are related to a perturbation of radial glial cells during development (Paredes et al., 2006). Developmental mechanisms appear different though from mouse models mentioned above, CA1 heterotopic hippocampal neurons were characterized as being derived from the neocortex and integrated in both neocortical and hippocampal circuitry (Chevassus-Au-Louis et al., 1998b,a; Paredes et al., 2006). Hippocampal cells have been shown to have altered glutamate receptor subunit and transporter expression and the affected animals to suffer from epilepsy (Harrington et al., 2007). This model may be useful for further studying structural and functional defects related to CA migration disorders.

## Temporal aspects of CA3 cell migration and the influence of mossy fibers—an analysis of further mutants

The migration process follows a temporal sequence that is likely to impact cell connectivity (Stanfield and Cowan, 1979a; Bayer, 1980; Deguchi et al., 2011). While pyramidal neurons migrate uniformly and synchronously through the cortical wall along glial fibers, it has been proposed that the long pausing of CA3 cells in the IZ may be due to a necessary synchronization with the development of mossy fibers (largely starting at E17–E18). Initial observations showed that a major proportion of DG granule cells are born later than CA3 cells, and the DG blades are established in early postnatal stages (Altman and Bayer, 1990a; reviewed in Danglot et al., 2006). On the other hand, Deguchi et al. (2011) using sparse Thy1-mGFP reporter lines, were also able to show that some DG cells are born as early as E12 in the mouse hippocampus and that there is selective connectivity (neuronal specification and synaptogenesis) between CA3 and DG cells, and CA3 and CA1 cells, born at the same times. These latter data suggest that synchronization of the production of different cell types occurs and that microcircuits may be important in the hippocampus.

Although the production of connecting granule cells may therefore be temporally correlated with CA3 cell partners (Deguchi et al., 2011), it is possible that the CA3 pyramidal layer can assemble correctly even if DG formation is perturbed. However, as mentioned above, there do exist mouse mutants where both the DG and CA pyramidal layers are disorganized, for example, *reelin* (Del Río et al., 1997; Lambert de Rouvroit and Goffinet, 1998) and *Lis*1 mutants (Fleck et al., 2000; Wang and Baraban, 2007, 2008). The *Girdin* mutant also, related to the Disc1 pathway, and *Grik2* “*weaver*” mutants show abnormal pathfinding of MFs and also some abnormal CA lamination or clusters of heterotopic cells (Sekiguchi et al., 1995; Enomoto et al., 2009). Differing from this, in the case of the *Cxcr*4 or *Disc*1 mouse mutants, granule cells are not guided properly to the DG region and remain accumulated in the migration path (Lu et al., 2002; Meyer and Morris, 2009; Kvajo et al., 2011), but in each case the pyramidal cell layer appears to form correctly, despite the granule cell abnormalities. It still remains possible on the other hand, that mossy fiber outgrowth occurs correctly in these mutants, despite abnormal granule cell position, hence still allowing for an effect of mossy fibers on CA3 cell migration. Conditional mutants are required to inactivate genes specifically in mossy fibers in order to verify pre-synaptic consequences on CA cell lamination.

A further set of mutants, with apparently correctly formed CA3 pyramidal and DG layers but more subtle perturbed mossy fiber targeting or pruning, are related to several axon guidance or adhesion molecules. For example inactivation of the semaphorin 3 receptors, *plexin* A3 and *neuropilin* 2 (Chen et al., 2000; Giger et al., 2000; Bagri et al., 2003), the guidance receptor *ephrin* B3 (Xu and Henkemeyer, 2009), and the adhesion molecule *nectin* (Honda et al., 2006), show mossy fibers which target infrapyramidal, as well as suprapyramidal regions, most probably due to abnormal fasciculation, targeting and pruning. Also, mouse mutants for *serum response factor* (Knöll et al., 2006), and adhesion molecules *Chl*1 (Montag-Sallaz et al., 2002), *Ncam*-180 (Seki and Rutishauser, 1998) and *cadherin* (Bekirov et al., 2008; Williams et al., 2011), have mossy fibers which aberrantly synapse on CA3 somata within the pyramidal cell layer instead of apical dendrites in the suprapyramidal regions. Furthermore, in mutants for semaphorin 6 receptors *plexin* A2 and A4, the mossy fibers also continue to project either to the infra- and/or intrapyramidal regions (Suto et al., 2007; Tawarayama et al., 2010). In some of these cases of aberrant intrapyramidal connections, the CA3c region appears thickened, but this may be due to the presence of excess neuropil within the layer, more so than abnormal migration, although this remains to be formally demonstrated. A different problem was revealed by mutation of the *grik2* gene, associated with autism spectrum disorder and intellectual disability, which slowed the structural and functional maturation of the hippocampal mossy fiber to CA3 pyramidal cell synapses (Lanore et al., 2012). These problems affecting mossy fiber fasciculation, targeting, synaptogenesis, and refinement do not hence answer the question if correct mossy fiber outgrowth and contact are strictly required for the final stages of CA3 cell migration and lamination, although this data seems to suggest that the migration of CA3 pyramidal neurons can occur relatively correctly, independently of mossy fibers. Specific bi-directional, trans-synaptic, interactions between DG and CA3 neurons remain however, critical to drive synapse formation.

## Electrophysiological, behavioral, and cognitive phenotypes

In all mouse mutants for genes associated with type I lissencephaly, or other similar cortical malformations (*Dcx*, *Kif2a*, *Tuba1a*, *Lis*1, *reelin*), a gradient of severity is observed at the functional and behavioral levels that parallels the gradient of severity that is observed at the cellular level. Consistent with an extensive neuronal disorganization in both the neocortex and hippocampus, *reeler* mice exhibit severe motor, behavioral, and emotional impairments (Falconer, 1951; Tueting et al., 1999, 2006; Gebhardt et al., 2002; Salinger et al., 2003). In the *Lis*1 model, the neuronal migration defects are associated with morphological and functional alterations of the Schaffer collateral–CA1 synapse and enhanced excitability in pyramidal neurons (Fleck et al., 2000; Jones and Baraban, 2007, 2009; Valdés-Sánchez et al., 2007; Greenwood et al., 2009). DG granule cells are also affected (Hunt et al., 2012). These mice exhibit spontaneous seizures (Hirotsune et al., 1998; Fleck et al., 2000; Jones and Baraban, 2009), as well as limited, albeit severe, cognitive deficits (Hirotsune et al., 1998; Lambert de Rouvroit and Goffinet, 1998; Paylor et al., 1999). Most studies which have been performed in *Lis*1 mutant mice have been focused on the CA1 region. Firstly, it was shown that CA1 heterotopic cells are more excitable compared to WT when hippocampal slices were subjected to increasingly elevated concentrations of potassium (Fleck et al., 2000). Single cell studies after stimulation of the Schaffer collaterals, further showed increased excitatory post-synaptic events (EPSPs) in mutant CA1 cells compared to WT. A laminar analysis of evoked EPSPs (with the recording electrode placed at different positions), showed that abnormally positioned heterotopic CA1 cells are also functionally innervated. Migration defects, EPSP abnormalities and some behavioral phenotypes were rescued by calpain inhibitors, which increase the quantities of Lis1 (Yamada et al., 2009; Sebe et al., 2013). Inhibitory post-synaptic currents (IPSCs) were recorded in CA1 cells and also found to be increased (Valdés-Sánchez et al., 2007). Furthermore, interneuron distribution and firing was abnormal in this model (Jones and Baraban, 2007, 2009). These combined data hence illustrate the multiple consequences on hippocampal circuits due to perturbed hippocampal development.

**Figure 3:**
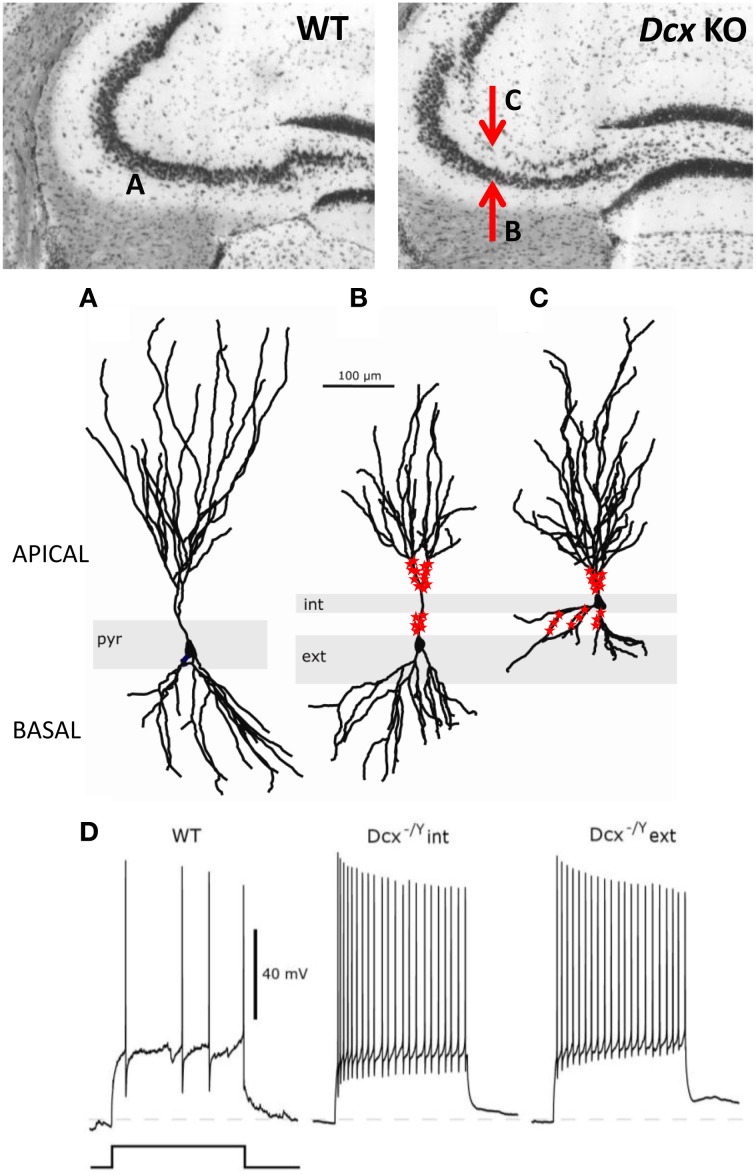
**Morphological and functional abnormalities in the *Dcx*-KO hippocampus.** Upper images show coronal slices of the adult *Dcx*-KO hippocampus (right) compared to wild-type (WT, left). Red arrows in the KO image point to the internal (upper) and external (lower) CA3 layers. Whole cell recordings and biocytin fills were performed to characterize internal (int) and external (ext) pyramidal cells. **(A)** WT cell; **(B)** KO external cell; **(C)** KO internal cell (taken and modified from Bazelot et al., 2012 Figure 5, published by John Wiley & Sons Ltd). Analyses of cell morphologies revealed reduced lengths of apical dendrites for external layer cells and reduced lengths of basal dendrites for internal layer cells (Bazelot et al., 2012). Red labeling on dendrites schematizes mossy fiber innervation in the form of post-synaptic thorny excrescence-like spines. Note non-continuous innervation on apical dendrites of external layer cells, and basal as well as apical innervation on internal layer cells (schematizing data presented in Bazelot et al., 2012, Figure 4). **(D)** Whole cell recordings taken from Bazelot et al. (2012) Figure 6 (published by John Wiley & Sons Ltd) revealed that KO cells are more excitable than their WT counterparts. In response to identical depolarizing current injection, KO cells fired at higher frequencies.

Unlike *Lis*1 mutants, *Dcx-*KO mice with cellular lamination defects largely restricted to the CA3 region, have provided a unique model to explore hippocampus-and CA3-dependent functions. At the clinical level, these mice exhibit spontaneous convulsive seizures and are more prone to seizures induced by chemoconvulsants than their WT littermates (Nosten-Bertrand et al., 2008). The precise anatomical characterization of the CA3 region revealed three distinct patterns of lamination in *Dcx*-KO mice along the rostro-caudal axis, with CA3b and c subregions exhibiting both double and dispersed layers of pyramidal cell somata, while the CA3a region was mostly a single layer (Bazelot et al., 2012; Germain et al., 2013). The density and distribution of parvalbumin-containing interneurons were not affected in *Dcx*-KO mice, and they were found to innervate pyramidal cell somata of both single and double (internal and external), layers. In addition, intracellular recordings, biocytin filling, as well as multi-electrode recordings showed that both layers of CA3 KO pyramidal cells exhibited an altered dendritic form and were more excitable than their WT counterparts (Bazelot et al., 2012). However, the bi-layer seems to have dramatic effects on pyramidal cell form, with the internal layer showing reduced basal dendrites, whereas the external layer showed reduced apical dendrites (Figure 3). Mossy fiber connections were also altered, related to the bi-layer, with some connections abnormally retained on the basal dendrites of the internal layer, whereas connections were interrupted on the apical dendrites of cells in the external layer, as they passed through the internal layer. The threshold current required to initiate an action potential was reduced in *Dcx*-KO cells, and mean firing rates were increased. Thus, the absence of Dcx affects synaptic connectivity and results in enhanced pyramidal cell excitability.

At the behavioral level, *Dcx*-KO mice were shown to be hyperactive in novel environments, less aggressive, and more ambidextrous than their WT littermates (Germain et al., 2013). Given its central position within the trisynaptic hippocampal pathway, the CA3 region may contribute to the prominent role of the hippocampus in episodic memory and spatial representation of the environment (Eichenbaum et al., 2012). However, interestingly, we have shown that *Dcx*-KO mice exhibit normal spatial learning and contextual or cued memory as tested in the Barnes or water mazes, and in the fear-conditioning paradigms. *Dcx*-KO mice also exhibited spared CA3-dependent cognitive functions such as rapid contextual representation, novelty detection, and one-trial short-term memory, tested with a large battery of tests such as fear conditioning, paired associate learning and object recognition (Germain et al., 2013). Strikingly, in another mouse model, the *Dcx* mutation was associated with lethality in hemizygous male mice, probably due to the different genetic backgrounds (Corbo et al., 2002). Interestingly however, both *Dcx* models are largely identical anatomically, with identical lamination defects largely restricted to the CA3 region. In addition, this study showed very mild behavioral deficits in *Dcx* heterozygous females, including reduced freezing in both cued and contextual fear conditioning tests, similarly identified in Germain et al. (2013), that could be indicative of an anxiety-related phenotype.

Thus, in *Dcx* mutants, the CA3 heterotopia is responsible for hyperexcitability and seizures, whereas hippocampal-dependent cognitive circuits may have at least partially adapted to the CA3 bi-layer. This differs from the situation in *Lis*1 mice and other mutants, with a more severely malformed hippocampus, hyperexcitability and perturbed cognitive functions. Although, a genetic redundancy underlying functional compensatory mechanisms between Dcx and its closest homologs Dclk1 and Dclk2 has been specifically addressed and demonstrated to be important in the neocortex and CA1 region of murine *Dcx* models (Deuel et al., 2006; Koizumi et al., 2006b; Kerjan et al., 2009), the CA3 anatomical defects are present in single *Dcx* knockouts. These observations again suggest more complex and specific features of cell migration in the CA3 region. The presence of Dclk1 and 2, and other potential compensatory proteins, in the developing CA3 region is not sufficient to allow correct neuronal migration and excitability, however the KO hippocampus still correctly performs hippocampus-dependent cognitive functions.

Two further mouse mutants showed mainly CA3 disorganization in the hippocampus, *Kif*2a and *14-3-3*ζ (Homma et al., 2003; Cheah et al., 2012) and it would hence be interesting to compare their behavioral phenotypes. Constitutive *Kif*2a mutants, have multiple brain abnormalities and die within a day of birth (Homma et al., 2003), however, *14-3-3*ζ mutants survive and exhibit striking behavioral deficits including hyperactivity, disrupted sensorimotor gating, and impaired learning and memory (Cheah et al., 2012). Some of these abnormalities are likely to be related to the hippocampal abnormalities. Notably though these mice also have developmental DG abnormalities, as well as severer abnormal mossy fiber targeting, differing hence from *Dcx*-KO mice.

## What have we learnt from studying the CA3 region in neuronal migration mutants?

1. The most striking observation from the murine models for genes involved in human neuronal migration disorders is that the CA3 region seems the most sensitive and consistently affected brain region. Indeed, all the mouse mutants for genes associated with type 1 lissencephaly, or other similar cortical malformations (*Lis1*, *Dcx*, *Tuba1a*, *reelin*, *Kif2a*), have a CA3 lamination defect as a common, and the most obvious, feature (Figure 2, Hirotsune et al., 1998; Lambert de Rouvroit and Goffinet, 1998; Fleck et al., 2000; Corbo et al., 2002; Homma et al., 2003; Kappeler et al., 2007; Keays et al., 2007; Poirier et al., 2013). Human patients also have severe hippocampal abnormalities, however, these are not restricted to the CA3 region. The mouse CA3 phenotype is also often associated with other variable defects. CA3 lamination defects also seem to cluster in some mouse mutants of genes associated with neuropsychiatric disorders (Enomoto et al., 2009; Petrone et al., 2003). Thus CA3 lamination abnormalities in the mouse are correlated with neurodevelopmental cortical problems in human, and models derived from large-scale ENU mutagenesis projects may be tested to search for CA3 migration defects in order to easily select interesting models for further exploration (Keays et al., 2007; Furuse et al., 2012).
  From a developmental point of view (section—Basic Steps of Hippocampal Development), it seems clear that CA3 cell migration is more complex than CA1. CA3 cells, especially in CA3b and c regions, have to migrate curved routes, which might involve several modes of migration, and particular features of radial glial cells that provide a cellular substrate. CA3 cell migration could hence require extra molecular and cellular pathways compared to CA1. The genes we mention in this review such as *Dcx*, are strictly necessary to achieve CA3 cell migration correctly and even close homologs cannot compensate their function. In genetic terms, there seems hence to be lower redundancy in the CA3 region. Further study of CA3 mutants will help to reveal the very particular aspects of CA3 development, compared to CA1. Some of these mechanisms may also be required for human neocortical development.
  It also remains possible that the CA3 region shows other susceptibilities. In the adult, CA3 cells are known to have a higher metabolic rate than CA1 cells, and to be more vulnerable to chronic stress and seizure-induced damages (Greene et al., 2009; Christian et al., 2011). Indeed, the CA3 is the most vulnerable subregion of the hippocampus in response to chronic stress and exhibits the most severe neuronal changes such as decrease in complexity and retraction of dendrites as compared to the CA1 subregion (Woolley et al., 1990; Watanabe et al., 1992). On the other hand, CA1 cells may be more susceptible to ischemia than CA3 pyramidal cells (Pulsinelli and Brierley, 1979; Zola-Morgan et al., 1986). CA3 cell organelle defects and increased cell death have also been identified in the *Dcx*-KO postnatal hippocampus (Khalaf-Nazzal et al., 2013). It is too early to tell if this may be a consequence of the aberrant migration, related to cell stress, or alternatively a consequence of the intrinsic function of Dcx, associated with MTs, which may affect organelle trafficking and subsequently migration. We cannot rule out however, that early metabolic or other differences during development also contribute to CA3 vs. CA1 migration susceptibilities.
2. Slowed and arrested migration in CA3 mutants clearly leads to a situation resembling heterotopia, with a small distance along the radial axis between different layers of cell somata. In this respect, it is interesting to recall, that phylogenetic differences in lamination have arisen, such that a bi-laminar CA1 region exists naturally in various mammalian species (Slomianka et al., 2011). Bi-lamination of hippocampal pyramidal cells *per se*, therefore is not necessarily expected to lead to aberrant functional consequences. It does however seem detrimental in CA3 mouse mutants, at least at the level of morphology, mossy fiber connectivity and potentially hyperexcitability (Kerjan et al., 2009; Bazelot et al., 2012; Cheah et al., 2012). Mossy fiber connectivity abnormalities seem to go together with abnormal lamination, even if the inverse is not always true (see discussion in section Temporal Aspects of CA3 Cell Migration and the Influence of Mossy Fibers—An Analysis of Further Mutants).
3. Further connectivity and functional studies still need to be performed, especially taking into account different interneuron populations, because interneuron migration abnormalities have also been identified in some mutants (*Lis*1, *Dcx*, *Dcx*/*Dclk*2, McManus et al., 2004; Kappeler et al., 2006; Kerjan et al., 2009). It is too early to tell if perturbed lamination contributes to abnormal positioning of hippocampal interneurons, even if the two processes may be coordinated (Manent et al., 2005; Lodato et al., 2011; Ciceri et al., 2013). During development, we and others have observed some interneuron and oligodendrocyte precursor cell disorganization within the developing CA3 layers (Kerjan et al., 2009; Khalaf-Nazzal et al., 2013). This may be related to a more diffuse organization of pyramidal cells (Khalaf-Nazzal et al., 2013).
  In the *Lis*1 model, as well as abnormal interneuron distribution, cell attached recordings showed that synaptic excitation of interneurons (EPSCs) was increased. IPSCs recorded in CA1 cells were also found to be increased (Jones and Baraban, 2007). The authors concluded that since precisely coordinated GABAergic activity is vital for the generation of oscillatory activity and place field precision in the hippocampus, these alterations in synaptic inhibition are likely to contribute to the seizures and the altered cognitive function observed. Loss of certain subpopulations is known to have a strong impact on susceptibility to epilepsy in other models (Cobos et al., 2005). Interneuron number and distribution can also change after seizures (Nosten-Bertrand et al., 2008; Kerjan et al., 2009). Interneuron function hence needs to be more closely examined in relation with CA3 phenotypes.
4. Concerning CA3-dependant cognitive function, it seems that it may be remarkably preserved in the *Dcx*-KO despite a bi-lamination of the CA3 pyramidal layer. However in this model, the synaptic excitatory and inhibitory input seem to be preserved in both CA3 layers (Bazelot et al., 2012), suggesting that despite the enhanced pyramidal cell excitability, synaptic transmission and plasticity could still be retained to an extent compatible with normal CA3-dependent function. Some previous studies have clearly shown intact spatial navigation despite focal CA3 impairment (Nakazawa et al., 2003; Nakashiba et al., 2008). In addition, several alternative systems may also adapt and compensate to maintain hippocampal function. Of interest, impaired CA3- and hippocampal cognitive function are reported when migration defects expand to other hippocampal subregions, such as the DG (*14-3-3*ζ mutants) or CA1 (*Lis*1 mutants).
5. CA3 deficits may be correlated with hyperexcitability and epilepsy, e.g., *Dcx* and *Lis*1 mutants, although defects in other cell types (e.g., interneurons), and in different hippocampal (e.g., CA1) and/or other brain regions (e.g., the neocortex) are very likely to also contribute to such a phenotype. Identifying the origins of hyperexcitability remains a complex subject, as does distinguishing between causative and neuroprotective mechanisms. Cataloguing the different mutants available for study and focusing on the most restricted phenotypes may help dissect out these pathogenic and pathophysiological mechanisms.

## Final conclusions

These fundamental studies concerning rodent models thus contribute to deepen our understanding of basic genetic and cellular mechanisms underlying lissencephaly and related disorders. They also shed light on specific CA3 molecular and cellular features, including more complex curved routes and longer pausing steps during cell migration, as well as a reduced potential of genetic and functional redundancy, that may all contribute to a higher vulnerability of this region. This may indeed extend to other human neurological and psychiatric disorders, for example different types of epilepsy and schizophrenia. In the past decade, thanks to rodent studies, the identification of cellular mechanisms underlying CA3 hyperactivity, including increased glutamatergic input from the amygdala, leading to CA3 interneuron loss, has significantly contributed to our understanding of psychotic-related symptoms such as social phobia and hallucinations (Liddle et al., 2000; Olypher et al., 2006; Lodge and Grace, 2007; Behrendt, 2010). Characterizing further these vulnerabilities will contribute to an improved pharmacological treatment of these morbid disorders in the future.

## Author contributions

Richard Belvindrah, Marika Nosten-Bertrand, and Fiona Francis wrote the review.

### Conflict of interest statement

The authors declare that the research was conducted in the absence of any commercial or financial relationships that could be construed as a potential conflict of interest.
